# In Vitro and Ex Vivo Antibiofilm Activity of a Lipopeptide Biosurfactant Produced by the Entomopathogenic *Beauveria bassiana* Strain against *Microsporum canis*

**DOI:** 10.3390/microorganisms8020232

**Published:** 2020-02-09

**Authors:** Marwa M. Abdel-Aziz, Mohsen S. Al-Omar, Hamdoon A. Mohammed, Tamer M. Emam

**Affiliations:** 1 Regional Center for Mycology and Biotechnology (RCMB), Al-Azhar University, Cairo 11371, Egypt; marwaemam.17@azhar.edu.eg; 2 Department of Medicinal Chemistry and Pharmacognosy, College of Pharmacy, Qassim University, Buraydah 51452, Saudi Arabia; M.OMAR@qu.edu.sa; 3 Department of Medicinal Chemistry and Pharmacognosy, College of Pharmacy, JUST, Irbid 22110, Jordan; 4 Department of Pharmacognosy, Faculty of Pharmacy, Al-Azhar University, Cairo 11371, Egypt; 5 Department of Microbiology, Desert Research Center, Buraydah 51452, Saudi Arabia; tameremam7@gmail.com

**Keywords:** dermatophytes, *Microsporum canis*, antibiofilm activity, ex vivo biofilm, biosurfactant, lipopeptide, entomopathogenic fungi

## Abstract

*Microsporum canis* is one of the most important dermatophyte causing tinea corporis and tinea capitis and its biofilm-form has a poor therapeutic response. The biosurfactant production by entomopathogenic fungi (EPF) has not been reported yet. The study aimed to investigate the potential usage of the EPF biosurfactant in the eradication of an ex vivo biofilm of *Microsporum canis* (*M. canis)* for the first time. An entomopathogenic fungus was isolated from the fungal-infected *Vespa orientalis* wasp and identified as *Beauveria bassiana* (MN173375). Chemical characterization revealed the lipopeptide nature of the *B. bassiana* biosurfactant (BBLP). Efficient antifungal and antibiofilm activities of BBLP against *M. canis* in vitro were detected. An ex vivo hair model was used to investigate the efficiency of BBLP against *M. canis* biofilm, in a scenario close to the in vivo conditions. *M. canis* ex vivo biofilm eradication was confirmed in stereo, scanning electron, and fluorescent images. Also, the ex vivo biofilm was less susceptible to BBLP treatment compared to its in vitro counterpart. In conclusion, BBLP showed significant eradication to the *M. canis* ex vivo biofilm and open horizons to use bio-resource derived from EPF in controlling microbial biofilm and holding great promise for combating recalcitrant dermatophytosis.

## 1. Introduction

Dermatophytes encompass a range of primary pathogenic fungi that infect hair, nails, and skin to obtain nutrients for their subsistence [1,2]. Dermatophytosis affects both immune-competent as well as immune-compromised people [3]. The high incidence of dermatomycosis, especially in patients with immunodeficiency, has led to a public health problem worldwide [4]. Moreover, host immunity status affects the disease consequences, which may vary from localized to invasive infection [5]. *Microsporum canis* is a ubiquitous zoophilic dermatophyte causing tinea capitis and tinea corporis in children and young adults, respectively [6]. Zoophilic dermatophytic species including *Microsporum canis (M. canis),* frequently induce inflammation of the infected skin tissues [7]. Resistance of the fungus to a particular drug may develop due to improper usage or dosing regimen [8]. Also, biofilm-forming ability is deemed to be one of the important virulence factors for *M*. *canis* and other fungi which have gained attention in recent years [9,10,11,12].

The diversity of the biosurfactants allows them to perform a variety of functions in the petrochemical, environmental, pharmaceutical, food, and agricultural industries [13,14]. The usage of chemically based surfactants in skin treatments is problematic due to their prospective risks of skin allergy and irritation [15]. On the other hand, low toxicity, high biodegradability, low critical micelle concentration (CMC), high surface activity [16,17,18,19,20], good compatibility with human skin, and low irritancy of biosurfactants [15,21], make them favorable for pharmaceutical and cosmetic formulations. Also, biosurfactants are well known for their efficiency as antimicrobial and anti-biofilm agents against single and multi-species biofilms [22].

Despite these advantages, there are restrictions on the industrial use of biosurfactants due to their higher production cost than synthetic surfactants [23]. This problem can be tackled in two ways: (i) minimizing the production cost by using agro-industrial wastes as substrates [24] and (ii) further investigation for microorganisms which have high production capacities for biosurfactants. Currently, agro-industrial substrates such as sugar cane molasses, wheat straw, rice straw, beet molasses, and corn steep liquor have been used for biosurfactant fabrication at the commercial stage [17,25,26]. In this study, the biosurfactant was produced using corn steep liquor (CSL) as a substrate. The replacement of expensive substrates by a cheap agro-industrial waste can reduce production cost and increase their competitiveness with other expensive substrates-based biosurfactants [17,27].

Since the last decade, increasing attention has been paid to the isolation of biosurfactant-producing organisms [28]. Fungi are preferable to bacteria due to the simpler removal of fungal cells during downstream steps [29]. Entomopathogenic fungi are natural control agents of insects that have mostly substituted chemical pesticides in the agricultural field [30]. Entomopathogenic fungi genera have an evolutionary ability that allows them to compete in the rhizosphere and adapted to high-stress environmental conditions of the soil [30,31,32]. The present study aims to isolate a biosurfactant derived from the entomopathogenic *Beauveria bassiana* for the first time, and to evaluate its in vitro and ex vivo anti-biofilm activities against *M. canis*.

## 2. Materials and Methods

### 2.1. Pathogens

#### 2.1.1. The Dermatophytic Fungus

*Microsporum canis* ATCC 36299 was provided by the American Type Culture Collection (ATCC), and was maintained on Sabouraud dextrose agar (SDA) plates.

#### 2.1.2. The Entomopathogenic Fungus (EPF)

Fungal-infected *Vespa orientalis* wasps were collected from the wasp-keeping section in Entomology Department -Desert Research Center, Cairo, Egypt. An entomopathogenic fungus (EPF) was isolated from an infected *Vespa orientalis* wasp on Sabouraud dextrose agar (Himedia), supplemented with 1% yeast extract (SDAY). The EPF was identified using molecular identification tools, based on internal transcribed spacer (ITS)-rDNA sequencing, utilizing the fungal universal primer set of ITS1 (5′-TCC-GTA-GGT-GAA-CCT-GCG-G-3′) and ITS4 (5′-TCC-TCC-GCT-TAT-TGA-TAT-GC-3′). The polymerase chain reaction (PCR) thermal cycling conditions were as follows: initial denaturation was performed at 95 °C for 10 min, denaturation at 95 °C for 30 s for 35 cycles, annealing at 57 °C for 1 min, extension at 72 °C for 1 min and final extension at 72 °C for 10 min. The PCR purification kit (Qiagen, Germany) was used to purify the PCR product following the manufacturer’s instructions by sequencing of the purified PCR product using an automatic sequencer (ABI Prism 377; Applied Biosystems, CA, USA). The NCBI-BLAST was used to analyze the sequence homologies (http://blast.ncbi.nlm.nih.gov/Blast.cgi). The ITS sequence of the isolated entomopathogen was compared to the sequences in the database to detect the DNA resemblances [33]. Bio Edit software was used to assess the alignment and molecular phylogeny.

### 2.2. Biosurfactant Production

The EPF was cultured in corn steep liquid (CSL) medium [27] with 10% CSL (CSL was kindly provided from the Mostorod Starch and Glucose Factory, Cairo, Egypt) as the substrate for biosurfactant production. The medium was supplemented with ferrous sulfate (FeSO_4_, 2.0 mM), magnesium sulfate (MgSO_4_, 0.8 mM) and manganese sulfate (MnSO_4_, 0.2 mM). 10^6^ colony forming units (CFU)/mL of the seven-day culture of the EPF was injected to 100 mL of the CSL medium in conical flasks. The EPF was allowed to grow up to 7 days at 25 °C at pH 7 and 200 rpm. After incubation, the supernatants (cell-free) were collected and adjusted to pH 2 using hydrochloric acid (6 M) to precipitate the biosurfactant which was collected as a pellet by centrifugation for 20 min at 4 °C and 9000 rpm. The crude biosurfactant was neutralized using 1 M NaOH. The neutralized precipitate was dialyzed against demineralized water in a Cellu-Sep© membrane (Seguin, USA) for 48 h [34] for purification.

### 2.3. Identification of the Biosurfactant

#### 2.3.1. Chemical Nature and Surface Active Properties of the Biosurfactant

Three reagents were used to identify the chemical nature of the biosurfactant in a qualitative manner according to the literature [35]. The chromogenic reagents: 0.5% ninhydrin solution in acetone, phenol-sulfuric acid solution (3% phenol and 5% sulfuric acid in absolute ethanol), and rhodamine 6G reagents were used to identify the peptides, carbohydrates, and lipids in the biosurfactant, respectively [35]. The produced Biosurfactant by *B. bassiana* is a lipopeptide and designated as BBLP. Thus, the total protein was estimated according to the pyrogallol red colorimetric total protein kit (SPINREACT, St. Esteve de Bas, Spain). The total lipid was estimated according to the sulfo-phosphovanillin colorimetric method using REactivos GPL total lipid kit (Spain). BBLP was subjected to surface-active screening assays such as emulsification index (EI24) [36], drop collapse [37], oil displacement [38], lipase activity [39], surface tension (ST) [40], and critical micelle concentration (CMC) [41].

#### 2.3.2. Structural Identification of the Biosurfactant

Chromatographic and spectroscopic analysis were used to elucidate the chemical composition of the biosurfactant. The purified biosurfactant was subjected to ^1^H NMR spectroscopy (Bruker AV600 NMR spectrometer, Germany). The biosurfactant sample was dissolved in deuterated DMSO and the chemical shifts were expressed in parts per million (ppm) down-fields from an internal standard of tetramethylsilane (TMS).

The biosurfactant was hydrolyzed with 6 mol/L HCl at 100 °C for 24 h, extracted with diethyl ether. The organic phase was dried with anhydrous Na_2_SO_4_ and evaporated under reduced pressure. The crude diethyl ether extract which contains the fatty acid components of the BBLP was methylated [42] and subjected to gas chromatography mass spectroscopy (GC–MS) analysis using a Thermo Trace GC Ultra coupled with Polaris Q MS and TriPlus auto-sampler using a DB-5 (0.25 mm × 30 m × 0.22 µm) column by passing helium as a carrier gas. The temperature was set between 60 °C to 260 °C and ramped at a rate of 10 ^°^C/min. The initial temperature was held for 2 min and at final temperature of 260 °C for 10 min. The GC flow rate was 1 mL min^−1^ and the total run time was 32 min. Mass was performed at scan mode between m/z 50–300. The temperature of the ion source was 200 °C. The mass spectra obtained for the fatty acid methyl esters were matched with the library of the National Institute of Standards and Technology (NIST) database.

Reversed phase high performance liquid chromatography (RP-HPLC) was used to identify the amino acids contents of the aqueous fraction. The amino acids of the BBLP as well as standard series of amino acids were subjected to derivatization with phenyl isothiocyanate [43]. The derivatized amino acid sample and standards were analyzed with RP-HPLC system which was equipped with a Shimadzu pump LC-103, and 5010 ultraviolet/visible (UV/VIS) Detector. Reversed phase C-18 column was used to separate amino acids which were detected by UV detector at 254 nm. The amino acid derivatives were separated using a multi-step linear gradient with two solvents; solvent A was 15.2 g of sodium acetate dissolved in 1850 mL of water (pH 6.5) and solvent B was composed of acetonitrile:distilled water (80:20, *v*/*v*). The gradient was run for 32 min and the flow rate was maintained at 1.0 mL/min.

Mass spectrum of the BBLP was carried out on the direct probe controller inlet part to single quadropole mass analyzer (thermo scientific gcms) model (isq lt) using Thermo x-calibur software (thermo scientific gcms) model (isq lt) using Thermo x-calibur software

### 2.4. Mycelial Growth Inhibition Assay

The BBLP was added to 90 mm diameter SDA plates and the final concentrations were adjusted to be 0.12, 0.24, 0.49, 0.98, 1.95, 3.9, and 7.81 μg/mL. The plates without BBLP treatment served as the control. Mycelial disks (5 mm) of *M. canis* (obtained from seven days old culture *M. canis*) were inserted to the center of SDA plats. The plates were incubated (25 °C) until *M. canis* growth in the control reaches the edge of the plates. The *M. canis* mycelial growth inhibition in the plates containing BBLP was compared to the control plates. The inhibition percentage was calculated using the equation:

(1) $$Inhibition\;\;\%=\left[\frac{C-T}{C}\right]\;\times \;100.$$

The *C* and *T* refer to the growth of *M. canis* (mm) in the control and treated plates. The minimum inhibitory concentration (MIC) was calculated from three intendant replicates as the lowest concentration required to completely inhibiting the *M. canis* growth.

### 2.5. Effect of *Beauveria Bassiana* Biosurfactant (BBLP) on Microsporum canis (M. canis) Biofilm

We inoculated 100 μL of *M. canis* suspension (1 × 10^6^ conidia/mL) in RPMI 1640, amended with fetal bovine serum, into 96-well microtiter plate and incubated for 48 h at 25 °C. After biofilm formation, the media and free-floating cells were removed; the biofilms were thoroughly washed in phosphate buffer saline (PBS) for three times. The multiple MIC-based concentrations of BBLP (the MIC, double, four, six, and eight folds of the MIC) were then added to the biofilms in 96-well plates and incubated for 24 h at 25 °C. BBLP-free wells were used as a control. The inhibitory effect of BBLP on *M. canis* biofilm was measured using the (2,3-bis (2 methoxy-4-nitro-5-sulfophenyl)-[(phenylamino)-carbonyl]-2H-tetrazolium hydroxide) (XTT)-reducing assay according to the reported method [44]. Briefly, the XTT solution was prepared by dissolving XTT [Sigma, St. Louis, USA], in the PBS at a concentration of 0.5 g/L. 100 μL of the XTT prepared solution was micropipetted to each well. The plates were then incubated for 2 h in the dark at 25 °C. The reduction in the XTT color intensity was measured spectrophotometrically in the microplate reader (BioTek, USA) at 492 nm. The percentage of biofilm eradication (%) was calculated from the following equation:

(2) $$Inhibitory\;\%\;of\;the\;M.\;canis\;biofilm=\left[1-\left(\frac{A}{A^{\prime }}\right)\right]\;\times \;100.$$

where *A* and *A’* were referred to the absorbance obtained from treated and control experiments at 492 nm, respectively. The minimum biofilm eradication concentration (MBEC) was calculated from three independent replicates [45].

### 2.6. M. canis Ex Vivo Biofilm

#### 2.6.1. Ex Vivo Biofilm Formation

The ex vivo experiment for the biofilm assay was conducted by the method mentioned in the literature [46], with some modifications; healthy hair strands were washed, cut into about 100 mg pieces and autoclaved (121 °C/15 min) to remove any surface contaminants that might affect the tests. They were then kept in sealed tubes at room temperature until use. At first, 2 mL of 1% bacteriological agar (Himedia) was inoculated in 24-well plate and solidified at 25 °C. Afterward, 50 mg of the pre-sterilized hair fragments were added to the wells containing the bacteriological agar. 100 μL of *M. canis* at 10^6^ CFU/mL was added to each well. The plate was incubated at 25 °C for seven days. The outer most wells were daily inoculated with 2 mL of sterile saline to prevent drying of the agar wells. After incubation, *M. canis ex vivo* biofilms were washed to remove non-adherent cells, transferred to new 24 well plates, and left to dry.

#### 2.6.2. BBLP Treatment of *M. canis* Ex Vivo Biofilm

*M. canis ex vivo* biofilms were exposed to MBEC, 2× MBEC, and 4× MBEC of BBLP in 12-well plate for 24 h. The *M. canis ex vivo* biofilms without BBLP was used as a control. After incubation, treated and untreated ex vivo biofilms were subjected to colony-forming unit counting and microscopic examination. Sterile water (200 μL) was added to *M. canis ex vivo* biofilms in a 12-well plate followed by vigorous washing to suspend the biofilm cells. The suspensions were then diluted to 1:100, after which half of the diluted suspensions were used for inoculating SDA plates for colony counting [12].

#### 2.6.3. Microscopic Observations of *M. canis* Ex Vivo Biofilms

Treated and untreated *M. canis ex vivo* biofilms were visualized using stereo microscopy, scanning electron microscopy (SEM), and fluorescence microscopy (FM). For the stereo microscope, ex vivo biofilms were observed with a Leica stereo Zoom Browser EX microscope and photographed with a Canon S50 digital camera. For SEM preparation, *M. canis ex vivo* biofilms were fixed in 3% glutaraldehyde followed by 1% osmium tetroxide for 2 h at room temperature. The samples were then dehydrated in a graded ethanol series followed by critical point dried and gold coated. The examination was performed using a JEOL JSM-5500LV at an accelerating voltage of 20 kV. For FM, ex vivo biofilms were stained with 50 mg/L propidium iodide (PI) for 30 min at 4 °C in the dark. Residual dye was removed by washing with phosphate-buffered saline. *Ex vivo* biofilms were examined by a Leica microscope (Wetzlar Microscope GmbH, DM5000 B, Germany) [44].

### 2.7. Statistical Analysis

Statistical analysis was performed using a commercially available software program (SPSS 18; SPSS, Chicago, IL, USA). Values were presented as mean and standard deviation (SD) and 95% confidence interval values. Data were evaluated for normality using the Kolmogorov-Smirnov test of normality. Analysis of variance (ANOVA) followed by Tukey’s post-hoc test was used for comparison. The level of significance was set at *p* ≤ 0.05.

## 3. Results

### 3.1. Identification of Isolated Entomopathogenic Strain

**Figure 1:**
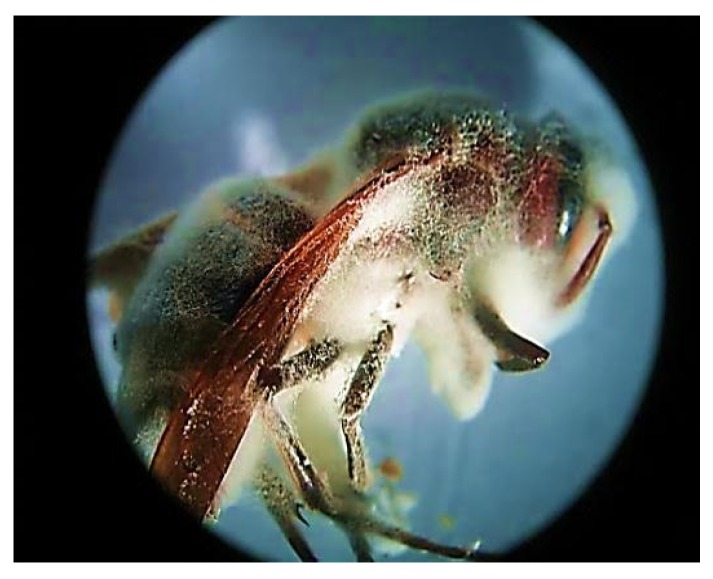
Stereo image of fungal infected *Vespa orientalis* wasp (2.5×).

**Figure 2:**
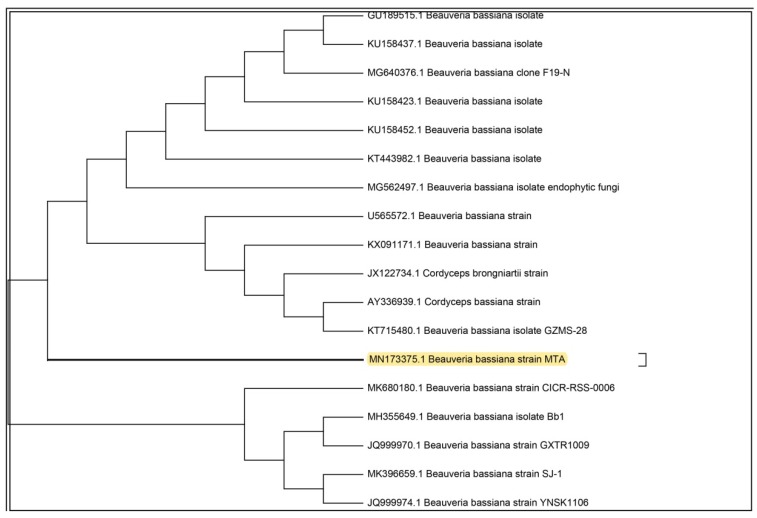
Phylogenetic tree of the isolated entomopathogenic *Beauveria bassiana.*

The entomopathogenic fungus (EPF), was isolated from an infected *Vespa orientalist* wasp (Figure 1) and was identified with the help of molecular tools. Upon molecular identification and phylogenetic analysis, the phylogenetic tree of the ITS rDNA sequence was constructed to illustrate its relationship with all sequences in the Gen-Bank database (http://blast.ncbi.nlm.nih.gov/). Accordingly, the ITS rDNA sequence obtained showed a homology of 99.74% with *Beauveria bassiana* (Figure 2) and was designated as *Beauveria bassiana* MN173375 (molecular analysis results are shown in the Supplementary Materials).

### 3.2. Structure Characterization and Surface Active Properties of BBLP

The lipopeptide nature of the produced biosurfactant by *Beauveria bassiana* was confirmed from the positive reaction with ninhydrin and rhodamine 6G reagents which demonstrated the presence of peptide and lipid portions in the biosurfactant molecule. Moreover, the chemical composition of the biosurfactant was quantitatively estimated and revealed that it is a mixture of protein and lipid in a combination of 78.35% and 21.65%, respectively. Furthermore, the BBLP exhibited remarkable results for all tested surface-active screening assays; with 92.4 ± 0.1% emulsification activity (Emulsification images are shown in the Supplementary Materials), positive activity in drop collapse, 112 U/mL lipase activity, and 12.65 ± 2.6 cm zone diameter in oil displacement (Oil displacement images are shown in the Supplementary Materials). Also, BBLP reduced the surface tension from 72 to 18 with 15 mg/L CMC.

**Figure 3:**
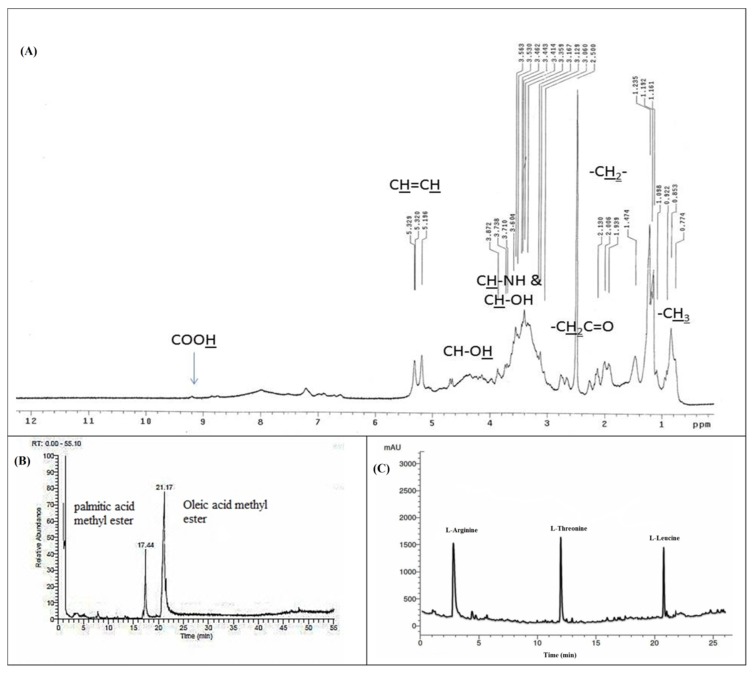
^1^H nuclear magnetic resonance (NMR) spectrum (**A**), GC-MS spectrum (**B**), and RP-HPLC spectrum of the *Beauveria bassiana* biosurfactant (BBLP) (**C**).

The lipopeptide nature of the BBLP was also confirmed by the ^1^H nuclear magnetic resonance (NMR) spectrum (Figure 3A) that revealed the presence of an overlapped long aliphatic chain (CH_2_) which observed at δ_H_ 1.09–2.12 ppm for the methylene aliphatic protons of the amino and fatty acids [43,47]. The presence of characteristic olefinic broad peaks of oleic acid were clearly observed at δ_H_ 5.19 and δ_H_ 5.32 ppm [47]. Furthermore, the peripheral methyl groups of the fatty acids and amino acids were also interfered at the δ_H_ 0.77 to δ_H_ 1.09 ppm. The hydroxyl protons (CH-OH) and carboxylic acid protons (COOH) of the amino acids were predicted downfield at δ_H_ 4.50 and δ_H_ 9.20 ppm. In addition, the absorption peaks of the amide hydrogen atoms were predicted between δ_H_ 7.05–8.00 ppm while the hydrogen atoms of the methylene protons which adjacent to the carbonyl moieties of the biosurfactant (CH_2_C=O) were predicted between δ_H_ 2.60 to δ_H_ 2.80 ppm in the ^1^HNMR spectrum of the BBLP compound [43] (Figure 3A).

The GC–MS analysis of the methyl esterified sample of the acid hydrolyzed organic fraction of the BBLP showed two major peaks at a retention time of 17.44, 21.17 min (Figure 3B). These peaks were identified as palmitic acid and oleic acid methyl esters based on the fragmentation pattern of the mass spectral data of the GC-MS and were consistent with the NIST database and literature [48]. The spectrum obtained from electron ionization mass spectrometry (EI-MS) showed the molecular ion peak for the BBLP at m/z 889.97 [M^+^]. The RP-HPLC analysis of the acid hydrolyzed aqueous fraction revealed the presence of three amino acids which matched with L-arginine, L-threonine, and L-leucine standards (Figure 3C).

### 3.3. In Vitro Susceptibility of M. canis Mycelial Growth and Preformed-Biofilm to BBLP

**Figure 4:**
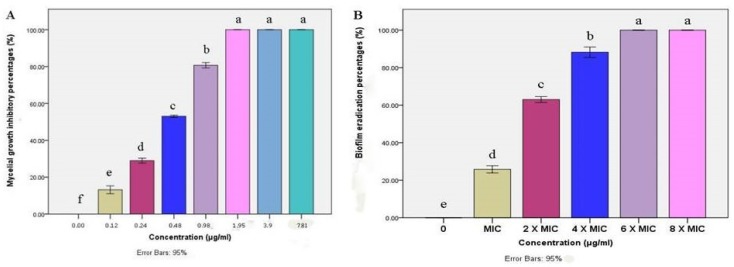
Bar chart showing (**A**) mean mycelial growth inhibitory percentages (%) of *M. canis* as affected by different concentrations (µg/mL) of BBLP; (**B**) *In vitro* biofilm eradication percentages (%) of *M. canis* as affected by different minimum inhibitory - based concentrations of BBLP; error bars 95%; Tukey’s post-test: bars sharing the same superscript letter are not significantly different.

The mycelial growth of *M. canis* was significantly inhibited by BBLP in a dose-dependent manner. Extremely significant differences (*p* < 0.0001) were demonstrated by ANOVA followed by Tukey’s post-hoc test between the concentrations of 0–1.95 µg/mL (Figure 4A). On the other hand, no significant differences between the concentrations of 1.95, 3.9 and 7.81 µg/mL. Besides, the *M. canis* mycelial growth was completely inhibited (MIC) at a concentration of 1.95 µg/mL. Moreover, the biofilm eradication percentage was increased by increasing the BBLP concentration. For instance, the biofilm eradication was 25.76% at the concentration of MIC (1.95 µg/mL), then the eradication was gradually increased to reach 100% at a concentration of 6× MIC (MBEC) (Figure 4B).

### 3.4. Obliteration of M. canis Ex Vivo Biofilms Resulted from BBLP Treatment

**Figure 5:**
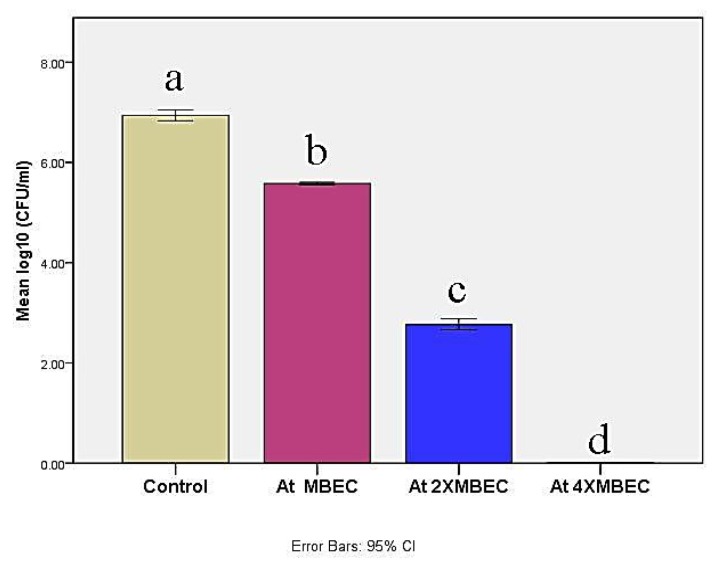
Bar chart illustrating mean log 10 (colony forming units (CFU)/mL) in control and at different concentrations of the BBLP. Significance level *p* ≤ 0.05, error bars 95%; Tukey’s post-test: bars with different superscript letter are significantly different.

Minimum biofilm eradication based concentrations exhibited log10 CFU significant reductions (*p* ≤ 0.05) against *M. canis ex vivo* biofilm in a concentration-dependent manner (Figure 5). MBEC, 2× MBEC of BBLP revealed CFU reduction by 1.36 log10 and 4.17 log10, respectively compared to control. Nevertheless, 4×MBEC of BBLP showed complete CFU inhibition (statistical analysis table is shown in the Supplementary Materials).

### 3.5. Microscopic Observations of M. canis Ex Vivo Biofilm

**Figure 6:**
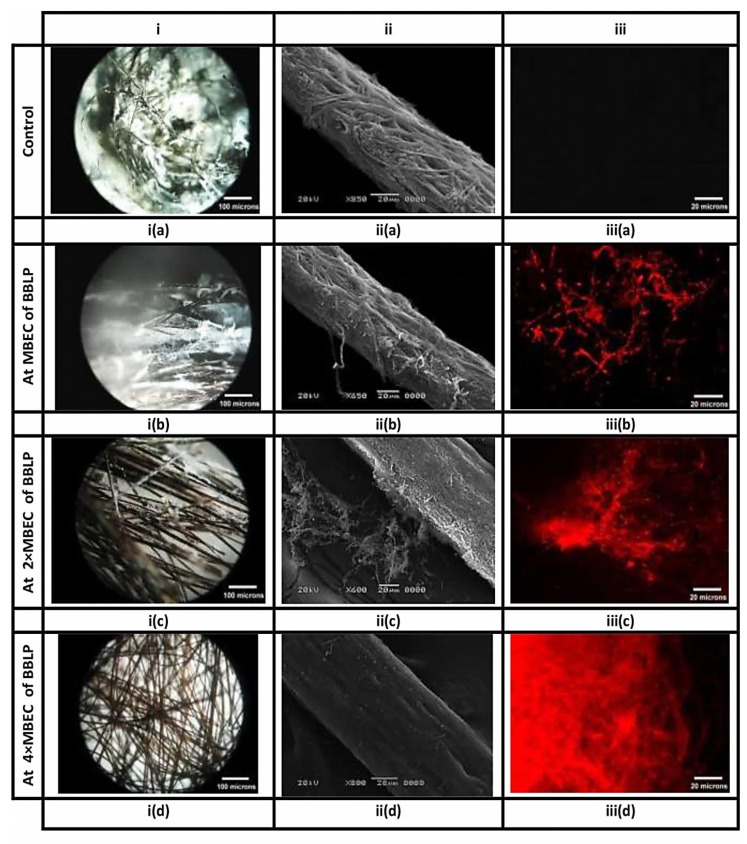
Biofilms of *M. canis*, formed on hair fragments, are visualized by different microscopic techniques. Columns (i), (ii) and (iii) correspond to stereo, scanning electron, and fluorescence images, respectively. Columns i and ii provide an overview and close up inspection of *M. canis ex vivo* biofilms, respectively, indicating the obliteration of the fungal elements, as they affected by BBLP in a dose-dependent manner (ia-iia, ib-iib, ic-iic, and id-iid, respectively) compared to the untreated controls (ia-iia) respectively. On the other hand, Column (iii) provides the appearance of red hyphae in an ascending order (iiib < iiic < iiid, respectively) indicating that the integrity of the cell membrane had been compromised as compared to the intact control without red hyphae (iiia).

*M. canis ex vivo* biofilm rapidly formed after 7d upon contact with the hair fragments, exhibited massive white fungal growth on the surface of hair fragments (Close up view of *M. canis ex vivo* biofilm is shown in the Supplementary Materials). *M. canis ex vivo* biofilms treated with MBE-based concentrations of BBLP and untreated ex vivo biofilms were submitted to stereo, scanning electron microscopic, and fluorescent microscope techniques (Figure 6). Stereo microscope, with low magnifications and oblique illumination, (Figure 6, column *i*) provided an excellent overview and preliminary inspection of *M. canis ex vivo* biofilms as affected by MBE-based concentrations of BBLP treatment, showing untreated *M. canis ex vivo* biofilm with dense white fungal growth (Figure 6 ia), which gradually decreased by increasing MBEC until it completely eradicated at 4× MBEC (Figure 6 ib, ic, id). On the other hand, SEM with a higher magnification and larger depth of focus, provided three-dimensional close up inspection of ex vivo biofilms, allowing a better assessment of the eradication of *M. canis ex vivo* biofilms as treated by MBE- based concentrations of BBLP (Figure 6, column ii); untreated *M. canis ex vivo* biofilm, exhibited dense mantel-like hyphae strongly attached to the hair fragment and the hair surface was completely replaced by fungal elements (Figure 6 iia). The eradication activity was in dose-dependent manner: 1) at MBEC of BBLP, few fungal elements were de-attached from the hair surface (Figure 6 iib); 2) at 2× MBEC of BBLP, higher amount of *M. canis ex vivo* biofilm shed from the hair surface with chunks of de-attached hyphae close to the hair strand were observed (Figure 6 iic), and 3) at 4× MIC, *M. canis ex vivo* biofilms was completely eradicated from the hair surface, leaving the hair topography with some erosions on the hair surface as the activity of *M. canis* during ex vivo biofilm maturation (Figure 6 iid). Regarding the FM observations, *M. canis ex vivo* biofilms displayed an increase diffusion of red PI staining pattern across the entire cell in a dose-dependent manner (Figure 6, column iii), which demonstrates that the BBLP treatment impacts the integrity of the *M. canis* cell membrane.

## 4. Discussion

Drug resistance of dermatophytes towards commonly used antifungal agents were reported [49]. Although *M. canis* is normally sensitive to antifungal drugs, however, biofilms are frequently resistant to standard antifungal agents [50]. Natural products have added many contributions to the health care system and improved human quality of life [51,52,53]. Drugs discovered from natural sources participated in treating complicated diseases and enriched the market with safe, effective, and renewable products [54]. However, it is a great challenge for scientists to find an effective natural product with minimal side effects [55]. In the current study, we hypothesized that entomopathogenic fungi could be a promising bio-source for biosurfactant production. Our hypotheses are based on two scientific facts; (i) entomopathogenic fungi (EPF) mainly depend on their enzymatic tools, consisting of lipase, protease, and chitinase, to establish their infection and lysis of the insect’s integument [31], (ii) lipase production quality is an important indicator for biosurfactant production by microorganisms as referred to by many earlier studies [39,56,57,58]. To test our hypothesis, we have isolated an entomopathogenic fungus from fungal-infected *Vespa orientalis* wasps. GenBank sequence database showed a 99.7% sequence identically with *Beauveria bassiana*. *B. bassiana* is known as an excellent entomopathogenic agent, which has been reported to infect more than 700 species of hosts belonging to many insect orders [30].

The chemical nature of the biosurfactant produced by *B. bassiana* (BBLP) is a mixture of lipid and protein (with approximately a 1:4 ratio) [39,59,60,61]. Impressively, the BBLP exhibited excellent surface active abilities which made it one of the rare fungal based-surfactants that can be effective in reducing surface tension, with respect to bacterial based-surfactants [14,23,62]. Moreover, the data obtained from GC-MS, RP-HPLC, and ^1^H NMR analysis ensure the lipopeptide nature of the BBLP and indicates the presence of oleic and palmitic acids as a lipid part; and L-arginine, L-threonine, and L-leucine as the protein part of the biosurfactant (Figure 3A–C). The mass fragmentation of oleic and palmitic acids in addition to the EI-MS spectrum of the BBLP (molecular ion peak m/z 889.97) confirm that the biosurfactant consists of only one unit from the previous mentioned amino acids linked together and with the fatty acids by amide linkages (mass spectra are shown in the Supplementary Materials).

The determination of antifungal activity revealed that BBLP has potent activity against *M. canis* mycelia growth, consistent with the previous reports of the lipopeptide biosurfactants potency as antifungal agents [60,61,62,63,64]. In addition, the MIC result (1.95 µg/mL) showed for BBLP was better than the results reported for the aqueous extract of 22 medicinal plants used as a remedy for the skin dermatophyte infections (MIC ranged from 3 to 35 µg/mL) [65]. Also, BBLP was more active than fluconazole standard antifungal drug which reported with MIC equal to 16 µg/mL against *M. canis* [66].

Until now, the antifungal activity against *M. canis* strains has been determined against their mycelial growth, which is unwarranted due to the ability of *M. canis* to form biofilm; this biofilm offers them protection against antibiotics and the host immune system and led to indolent infections [60]. In the current study, BBLP displayed significant antibiofilm activity against *M. canis* pre-formed biofilm. The results were consistent with the reported activity of battacin lipopeptides against planktonic and mature biofilms of *Candida albicans* [67]. One important observation during the study was that, BBLP has completely inhibited the growth of *M. canis* mycelial at a concentration of 1.95 µg/mL while the activity of BBLP was not more than 25.76 % against the *M. canis* established biofilms in the in vitro microplate assay. These findings showed that *M. canis* is more resistant when grown as biofilm rather than in planktonic form.

Many earlier studies revealed that lipopeptide biosurfactants can be a useful approach to challenge microorganisms growing as a biofilm [67,68,69,70]. Dermatophytes grow in the form of a biofilm in vivo, making them difficult to remove surgically and resistant to traditional therapies [50]. So, we think that it is of keen interest to investigate the inhibition of *M. canis* mature biofilm in an ex vivo model as a keratin substrate to detect the efficacy of BBLP in a scenario close to the in vivo condition. Using an ex vivo pattern, in which tissues or organs are extracted from an organism and placed in an artificial environment, is midway between the in vitro and the in vivo experiments. Since the *M. canis* is a causal agent of tinea capitis in humans and animals worldwide [5], thus, hair fragments were used as a host which resulted in a prompt adaptability of the *M. canis* to the host as compared with the other keratinized tissues and, subsequently, *M. canis ex vivo* biofilm has grown rapidly and formed on the hair surface. The stereo and scanning electron images revealed, for the first time, relevant aspects of the ex vivo biofilm dislodging process. Interestingly, complete eradication of *M. canis ex vivo* biofilm was observed at higher minimal eradication concentrations (MBECs), which pointed out that, *M. canis ex vivo* biofilm is less susceptible to BBLP treatment rather than its in vitro biofilm counterpart. These results could be explained by the ability of *M. canis* to form a more robust biofilm in an ex vivo condition rather than an in vitro condition [46]. The fluorescence microscopy (FM) observations revealed that BBLP interfered with the *M. canis* cell membrane integrity which is consistent with the reported effect of the lipopeptide biosurfactants on the permeability of the microbial cell membrane [71].

## 5. Conclusions

Biofilms of dermatophytes are resistant to conventional antifungal therapy; thus, there is an urgent need to employ innovative therapies to succeed in eradicating these microorganisms. There are few studies on the production of biosurfactants by filamentous fungi. This study presented a lipopeptide biosurfactant derived from an entomopathogenic fungus, for the first time. The entomopathogenic fungus (*Beauveria bassiana)*, showed an excellent ability to use agro-industrial substrate to produce high-value biosurfactant. Concerning dermatophytes, antibiofilm activity in vitro and ex vivo, are complementary techniques, useful for ensuring that the antibiofilm relevant data are consistent with that expected under in vivo conditions. BBLP eradicated the *M. canis ex vivo* biofilm and opens potential avenues to use biosurfactants from EPF in controlling microbial biofilm and holding great promise for combating recalcitrant dermatophytosis.

## Supplementary Materials

Supplementary materials can be found at https://www.mdpi.com/2076-2607/8/2/232/s1.
