# Supplementary material for: In Vitro and Ex Vivo Antibiofilm Activity of a Lipopeptide Biosurfactant Produced by the Entomopathogenic Beauveria bassiana Strain against Microsporum canis
Source: Microorganisms. 2020 Feb 9;8(2):232. doi: 10.3390/microorganisms8020232 (PMC7074774; doi:10.3390/microorganisms8020232)
Supplement: Supplementary file 1 [file microorganisms-08-00232-s001.pdf]

In vitro and *ex vivo* antibiofilm activity of a lipopeptide biosurfactant produced by the entomopathogenic- *Beauveria bassiana* strain against *Microsporum canis*

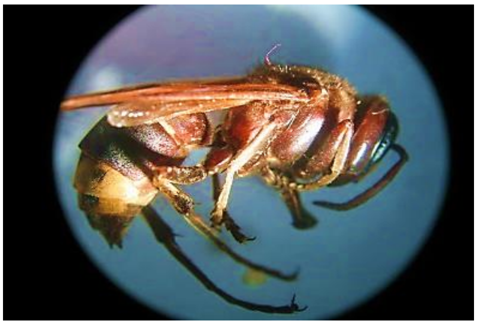

Figure S1: fungal free- *Vespa orientalis* wasp

	Description	Max Score	Total Score	Query Cover	E value	Per. Ident
✓	Beauveria bassiana isolate Bb1 small subunit ribosomal RNA gene, partial sequence; internal transcribed spacer 1, 5.8S ribosomal RNA gene, anc	699	699	97%	0.0	99.74%
✓	Cordyceps bassiana strain Bb126 small subunit ribosomal RNA gene, partial sequence; internal transcribed spacer 1, 5.8S ribosomal RNA gene a	699	699	97%	0.0	99.74%
✓	Beauveria bassiana strain CICR-RSS-0006 small subunit ribosomal RNA gene, partial sequence; internal transcribed spacer 1 and 5.8S ribosomal	697	697	97%	0.0	99.74%
✓	Beauveria bassiana strain SJ-1 small subunit ribosomal RNA gene, partial sequence; internal transcribed spacer 1, 5.8S ribosomal RNA gene, and	697	697	97%	0.0	99.74%
✓	Beauveria bassiana isolate endophytic fungi small subunit ribosomal RNA gene, partial sequence; internal transcribed spacer 1, 5.8S ribosomal RI	697	697	97%	0.0	99.74%
✓	Beauveria bassiana strain YNSK1106 18S ribosomal RNA gene, partial sequence; internal transcribed spacer 1, 5.8S ribosomal RNA gene, and int	697	697	97%	0.0	99.74%
✓	Beauveria bassiana isolate B4 18S ribosomal RNA gene, partial sequence; internal transcribed spacer 1, 5.8S ribosomal RNA gene, and internal tr	697	697	97%	0.0	99.74%
✓	Beauveria bassiana clone F19-N small subunit ribosomal RNA gene, partial sequence; internal transcribed spacer 1, 5.8S ribosomal RNA gene, an	695	695	97%	0.0	99.74%

Beauveria bassiana isolate Bb1 small subunit ribosomal RNA gene, partial sequence; internal transcribed spacer 1, 5.8S ribo gene, and internal transcribed spacer 2, complete sequence; and large subunit ribosomal RNA gene, partial sequence

Sequence ID: MH355649.1 **Length: 543** **Number of Matches: 1**

Range 1: 1 to 381 GenBank Graphics ▼ Next Match ▲ Previous Match

Score	Expect	Identities	Gaps	Strand
699 bits(378)	0.0	380/381(99%)	0/381(0%)	Plus/Minus

```
Query  7    CCGACCTCCCCAAGGGGAGGTCGAGGGTTGAAATGACGCTCGAACAGGCATGCCCGCCAG  66
            ||||||||||||||||||||||||||||||||||||||||||||||||||||||||||||
Sbjct  381  CCGACCTCCCCAAGGGGAGGTCGAGGGTTGAAATGACGCTCGAACAGGCATGCCCGCCAG  322

Query  67   AATGCTGGCGGGCGCAATGTGCGTTCAAAGATTCGATGATTCACTGGATTCTGCAATTCA  126
            ||||||||||||||||||||||||||||||||||||||||||||||||||||||||||||
Sbjct  321  AATGCTGGCGGGCGCAATGTGCGTTCAAAGATTCGATGATTCACTGGATTCTGCAATTCA  262

Query  127  CATTACTTATCGCGTTTCGCTGCGTTCTTCATCGATGCCAGAGCCAAGAGATCCGTTGTT  186
            ||||||||||||||||||||||||||||||||||||||||||||||||||||||||||||
Sbjct  261  CATTACTTATCGCGTTTCGCTGCGTTCTTCATCGATGCCAGAGCCAAGAGATCCGTTGTT  202

Query  187  GAAAGTTTTGATTCATTTGTTTTGCCTTGCGGCGTATTCAGAAGATGCTGGAATACAAGA  246
            ||||||||||||||||||||||||||||||||||||||||||||||||||||||||||||
Sbjct  201  GAAAGTTTTGATTCATTTGTTTTGCCTTGCGGCGTATTCAGAAGATGCTGGAATACAAGA  142

Query  247  GTTTGAGGTCCCCGGCGGGCCGCTGGTCCAGTCCGCGTCCGGGCTGGGGCGAGTCCGCCG  306
            ||||||||||||||||||||||||||||||||||||||||||||||||||||||||||||
Sbjct  141  GTTTGAGGTCCCCGGCGGGCCGCTGGTCCAGTCCGCGTCCGGGCTGGGGCGAGTCCGCCG  82

Query  307  AAGCAACGATAGGTAGGTTCACAGAAGGGTTAGGGAGTTGAAAACTCGGTAATGATCCCT  366
            ||||||||||||||||||||||||||||||||||||||||||||||||||||||||||||
Sbjct  [illegible]
```

Figure S2 Alignment and DNA sequence of *Beauveria bassiana*

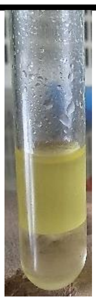

a

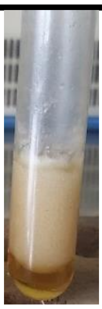

b

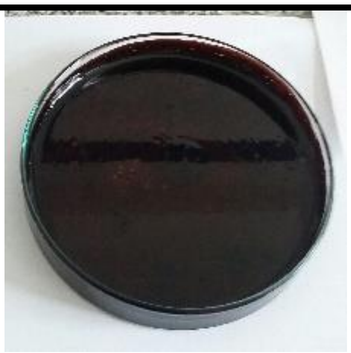

c

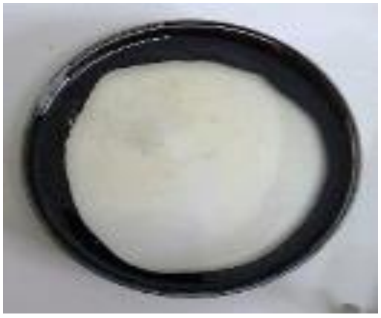

d

Figure S3. Emulsification index test (EI24) (a) control, (b) with BBLP; oil displacement test (c) control, (d) with BBLP.

Statistical analysis tables:

Table (S1) Comparison of Mycelial growth inhibitory percentages (%) of *M. canis* as affected by BBLP (ANOVA test)

BBLP conc (μg/ ml)	Mean	Std. Dev	Std. Error	95% Confidence Interval for Mean		Min	Max	F	P
				Lower Bound	Upper Bound				
0.00	0.00[f]	0.00	0.00	0.00	0.00	0.00	0.00	28589.4	<0.0001*
0.12	13.14[e]	0.86	0.50	11.00	15.29	12.38	14.08		
0.24	28.96[d]	0.54	0.31	27.62	30.30	28.35	29.37		
0.49	53.03[c]	0.24	0.14	52.42	53.63	52.76	53.24		
0.98	80.69[b]	0.59	0.34	79.22	82.15	80.22	81.35		
1.95	100[a]	0.00	0.00	100.00	100.00	100.00	100.00		
3.90	100[a]	0.00	0.00	100.00	100.00	100.00	100.00		
7.81	100 [a]	0.00	0.00	100.00	100.00	100.00	100.00		

Significance level p<0.05, *significant
Tukey's post test: Means sharing the same superscript letter are not significantly different

Table (2) In vitro Biofilm eradication percentages (%) of M. canis as affected by BBLP (ANOVA test)

BBLP conc (μg/ ml)	Mean	Std. Dev	Std. Error	95% Confidence Interval for Mean		Min	Max	F	P
				Lower Bound	Upper Bound				
0	0.00[e]	0.00	0.00	0.00	0.00	0.00	0.00	13963.03	<0.0001*
MIC	25.76[d]	.77	0.44	23.85	27.67	24.89	26.35		
2 ×MIC	63.01[c]	.63	0.37	61.44	64.59	62.58	63.74		
4 ×MIC	88.22[b]	1.12	0.65	85.44	91.01	87.25	89.45		
6 ×MIC	100[a]	0.00	0.00	100.00	100.00	100.00	100.00		
8 ×MIC	100[a]	0.00	0.00	100.00	100.00	100.00	100.00		

Significance level p<0.05, *significant
Tukey's post test: Means sharing the same superscript letter are not significantly different

Table (S3) Descriptive statistics of log 10 (CFU/ml) and comparison of different BBLP MBE-based concentrations (ANOVA test)

	Mean	Std. Dev	Std. Error	95% Confidence Interval for Mean		Min	Max	F	P
				Lower Bound	Upper Bound				
Control	6.94[a]	0.04	0.03	6.83	7.05	6.91	6.99	29258.3	0.05*
At MBEC	5.58[b]	0.01	0.01	5.56	5.60	5.57	5.59		
At 2XMBEC	2.77[c]	0.04	0.03	2.66	2.88	2.72	2.80		
At 4XMBEC	0.00[d]	0.00	0.00	0.00	0.00	0.00	0.00		

Significance level p≤0.05, * significant

Tukey's Post hoc test: means with different superscript letters are significantly different

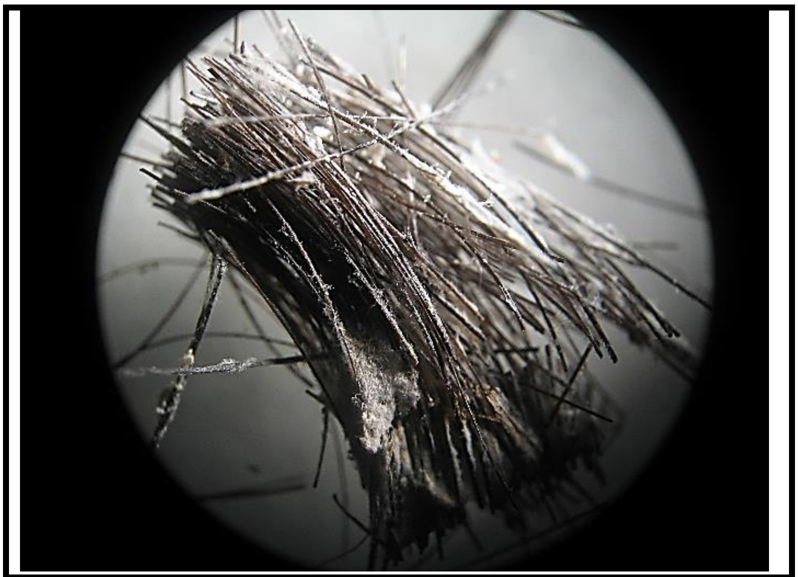

a

Figure S4. Close up view of *M. canis* ex- vivo biofilm

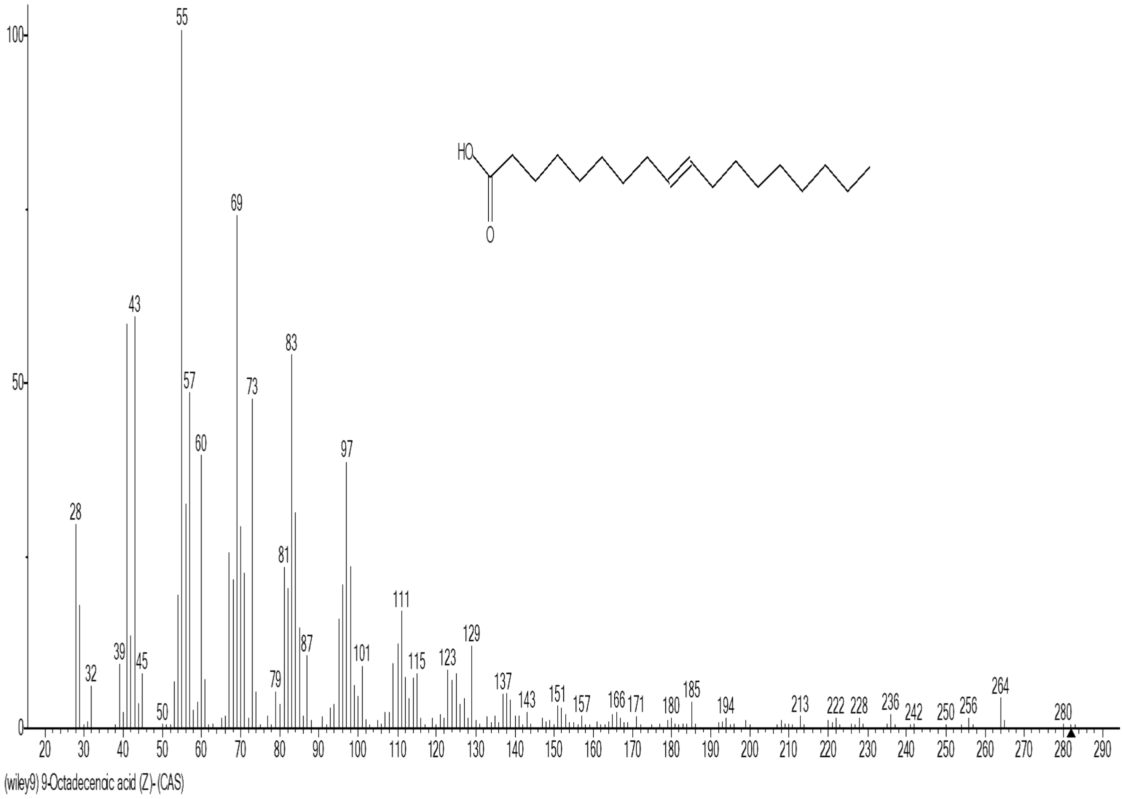

Figure S5. Mass fragmentation spectrum of oleic acid as obtained from the GC-MS analysis

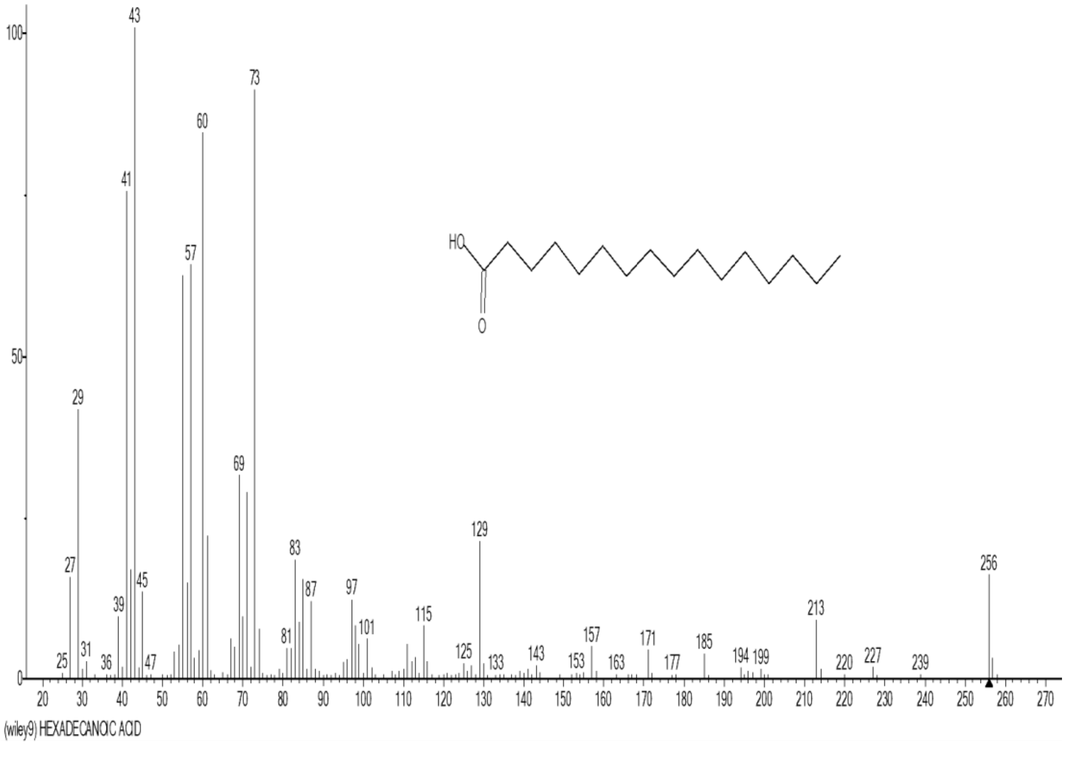

Figure S6. Mass fragmentation spectrum of palmitic acid as obtained from the GC-MS analysis

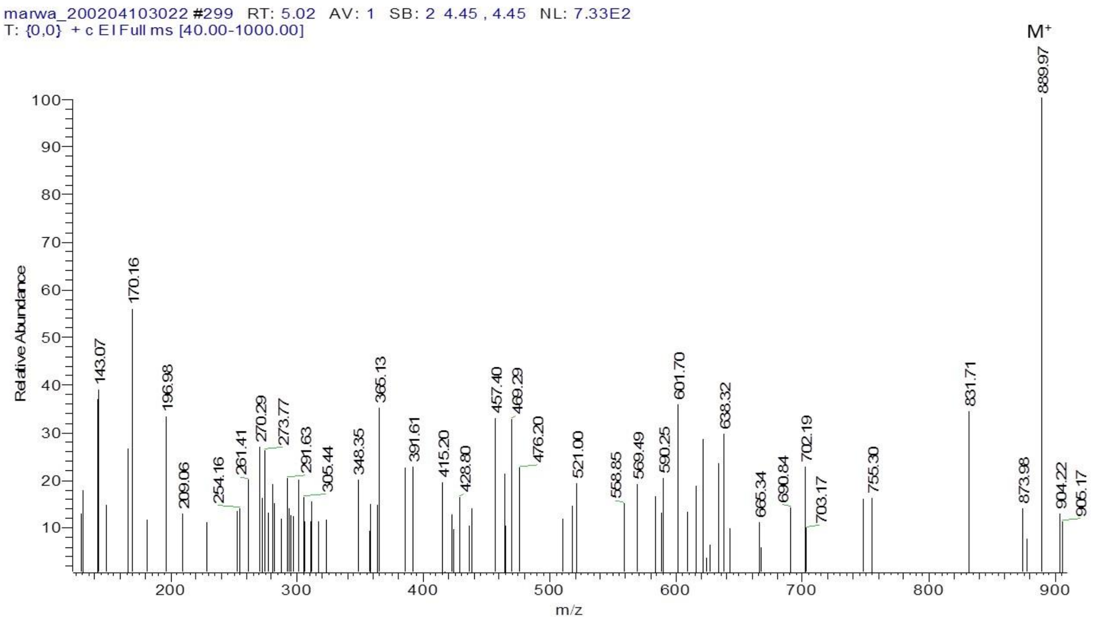

Figure S7. EI-MS of BBLP
